# Predicting efficacy of epirubicin by a multigene assay in advanced breast cancer within a Danish Breast Cancer Cooperative Group (DBCG) cohort: a retrospective-prospective blinded study

**DOI:** 10.1007/s10549-018-4918-4

**Published:** 2018-08-11

**Authors:** Anna Sofie Kappel Buhl, Troels Dreier Christensen, Ib Jarle Christensen, Knud Mejer Nelausen, Eva Balslev, Ann Søegaard Knoop, Eva Harder Brix, Else Svensson, Vesna Glavicic, Adam Luczak, Sven Tyge Langkjer, Søren Linnet, Erik Hugger Jakobsen, Jurij Bogovic, Bent Ejlertsen, Annie Rasmussen, Anker Hansen, Steen Knudsen, Dorte Nielsen, Peter Buhl Jensen

**Affiliations:** 1 Department of Oncology, Herlev and Gentofte Hospital, Copenhagen University Hospital, Herlev Ringvej 75, 2730 Herlev, Denmark; 2 Department of Pathology, Herlev and Gentofte Hospital, Copenhagen University Hospital, Herlev, Denmark; 3 Department of Oncology, Rigshospitalet, Copenhagen University Hospital, Copenhagen, Denmark; 4 Department of Oncology, Nordsjaellands Hospital, Copenhagen University Hospital, Hilleroed, Denmark; 5 Department of Oncology, Zealand University Hospital, Roskilde, Naestved, Denmark; 6 Department of Oncology, Aalborg University Hospital, Aalborg, Denmark; 7 Department of Oncology, Aarhus University Hospital, Aarhus, Denmark; 8 Department of Oncology, Regional Hospital West Jutland, Herning, Denmark; 9 Department of Oncology, Vejle Sygehus, Vejle, Denmark; 10 Department of Oncology, Hospital of Southern Jutland, Soenderborg, Denmark; 11 The Danish Breast Cancer Cooperative Group, DBCG Secretariat, Rigshospitalet, Copenhagen, Denmark; 12 Medical Prognosis Institute, Hoersholm, Denmark

**Keywords:** Epirubicin, Advanced breast cancer, Precision medicine, Predictive biomarker

## Abstract

**Purpose:**

Anthracyclines remain a cornerstone in the treatment of primary and advanced breast cancer (BC). This study has evaluated the predictive value of a multigene mRNA-based drug response predictor (DRP) in the treatment of advanced BC with epirubicin. The DRP is a mathematical method combining in vitro sensitivity and gene expression with clinical genetic information from > 3000 clinical tumor samples.

**Methods:**

From a DBCG cohort, 140 consecutive patients were treated with epirubicin between May 1997 and November 2016. After patient informed consent, mRNA was isolated from archival formalin-fixed paraffin-embedded primary breast tumor tissue and analyzed using Affymetrix arrays. Using time to progression (TTP) as primary endpoint, the efficacy of epirubicin was analyzed according to DRP combined with clinicopathological data collected retrospectively from patients’ medical records. Statistical analysis was done using Cox proportional hazards model stratified by treatment line.

**Results:**

Median TTP was 9.3 months. The DRP was significantly associated to TTP (*P* = 0.03). The hazard ratio for DRP scores differing by 50 percentage points was 0.55 (95% CI –0.93, one-sided). A 75% DRP was associated with a median TTP of 13 months compared to 7 months following a 25% DRP. Multivariate analysis showed that DRP was independent of age and number of metastases.

**Conclusion:**

The current study prospectively validates the predictive capability of DRP regarding epirubicin previously shown retrospectively allowing the patients predicted to be poor responders to choose more effective alternatives. Randomized prospective studies are needed to demonstrate if such an approach will lead to increased overall survival.

## Introduction

Breast cancer (BC) is one of the most common cancers worldwide and accounts for 15% of all cancer-related deaths among females [1]. Close to 20% of patients experience recurrence either as loco-regional or distant disease [2] in addition to the < 10% having primary advanced disease at the time of diagnosis [3]. Most of these patients are considered non-curable and treatment is limited to a palliative focus [3].

Anthracyclines, e.g., epirubicin, doxorubicin, and pegylated doxorubicin, are widely used in the different settings of BC treatment, and are in the (neo)adjuvant setting considered standard treatment [4]. In addition, anthracyclines are recommended in locally advanced or metastatic disease [5]. Doxorubicin is commonly used in the US, whereas the use of epirubicin is more widespread in Europe [6]. Epirubicin and doxorubicin are molecular alike with similar efficacy although epirubicin potentially has a better toxicity profile particular concerning cardiotoxicity [7].

Despite an increasing number of effective anticancer treatments, drug resistance is still a major concern resulting in treatment failure [8]. The efficacy of anthracyclines appears highly variable with response rates of 42–79% [9, 10]. Evidently a large proportion of patients do not obtain any benefit from the treatment but nonetheless experience adverse effects, and furthermore, initiation of a more effective treatment is delayed. When first-line treatment fails, it is well known that the benefit of second-line treatment and beyond becomes even more challenging [3, 11]. Systems to match a patient and a drug are eagerly awaited [12]. Thus, preferably each patients’ tumor should be evaluated in order to identify drugs most likely to have an effect in the individual patient.

**Fig. 1:**
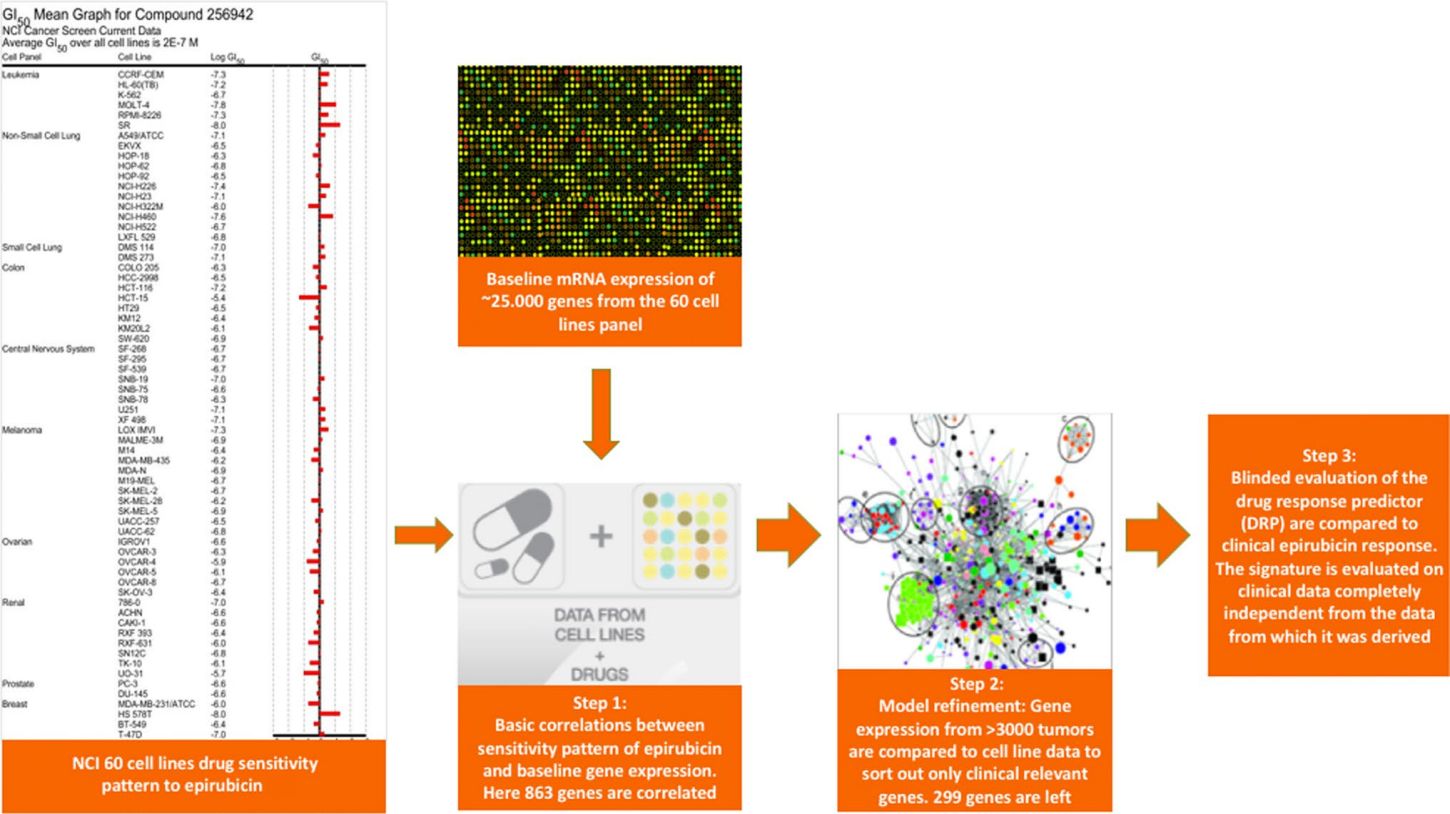
The principle behind the drug response prediction method

Medical Prognosis Institute has invented a cell line and multigene mRNA-based Drug Response Predictor (DRP) which is based on drug-specific genetic response profiles and evaluates a specific tumor’s potential response to a drug. The DRP algorithm is based on cell line data from National Cancer Institute (NCI) 60 cell line panel [13]. Available data include gene expressions from untreated cell lines. Likewise, GI50 values (drug dose that result in 50% reduction in growth) are present for different drugs and are regarded as measures of cell line sensitivity. In this way, sensitivity and resistance patterns from cell lines treated with the particular drug in vitro are available in a public domain. Gene expression from the untreated cell lines is correlated to the sensitivity pattern of the drug to show which genes are correlated to sensitivity and which genes are correlated to resistance in vitro. In order to only include the clinical relevant pathways, gene expressions from more than 3000 patients’ tumors of different origins are compared to the raw DRP. Gene expression that was not participating in any meaningful biological pathway is excluded from the final DRP. This method has increased the signal-to-noise ratio and is explained visually in Fig. 1.

The DRP has shown promise in several cancer diseases, drugs, and drug combinations and has been retrospectively validated in several settings [14–16]. Epirubicin as neoadjuvant monotherapy has been evaluated retrospectively by the predictor in 120 patients with early BC [17]. Further, as part of R-CHOP regime, doxorubicin has been validated in Diffuse Large B-Cell Lymphoma with the DRP using micro-RNA [18]. The aim of the current study was to evaluate the DRP among epirubicin-treated advanced BC patients.

## Methods

### Study design and patients

The current epirubicin cohort was identified among patients who were screened for participation in a phase 1 trial with liposomal cisplatin (LiPlaCis) [19].

A total of 1199 consecutive patients with locally advanced or metastatic BC were enrolled for screening in the LiPlaCis cohort. Inclusion criteria were patients with age ≥ 18 years with histological confirmed locally advanced or metastatic adenocarcinoma of the breast, expected life time ≥ 3 months, ECOG performance status (PS) ≤ 2, and written informed consent. Exclusion criteria were patients with other primary malignancy within the last 5 years prior to enrolment, except for adequately treated carcinoma in situ of the cervix, squamous carcinoma of the skin, or adequately controlled limited basal cell skin cancer. Patients were also excluded if they had any other disease or physiological dysfunction giving reasonable suspicion of a disease or condition that contraindicated the use of the investigational drug or place the patient at high risk from treatment-related complications. Tumor tissue was requested from the local pathology department and sent to the Coordinating Department of Pathology, Herlev and Gentofte Hospital where a subsample with high tumor cell content was selected prior to DRP analysis.

The participating sites were oncology departments at 10 Danish hospitals (Herlev and Gentofte, Herning, Hilleroed, Naestved, Rigshospitalet, Roskilde, Soenderborg, Vejle, Aalborg and Aarhus).

The study commenced in March 2013 and is ongoing.

#### Epirubicin prediction study

**Fig. 2:**
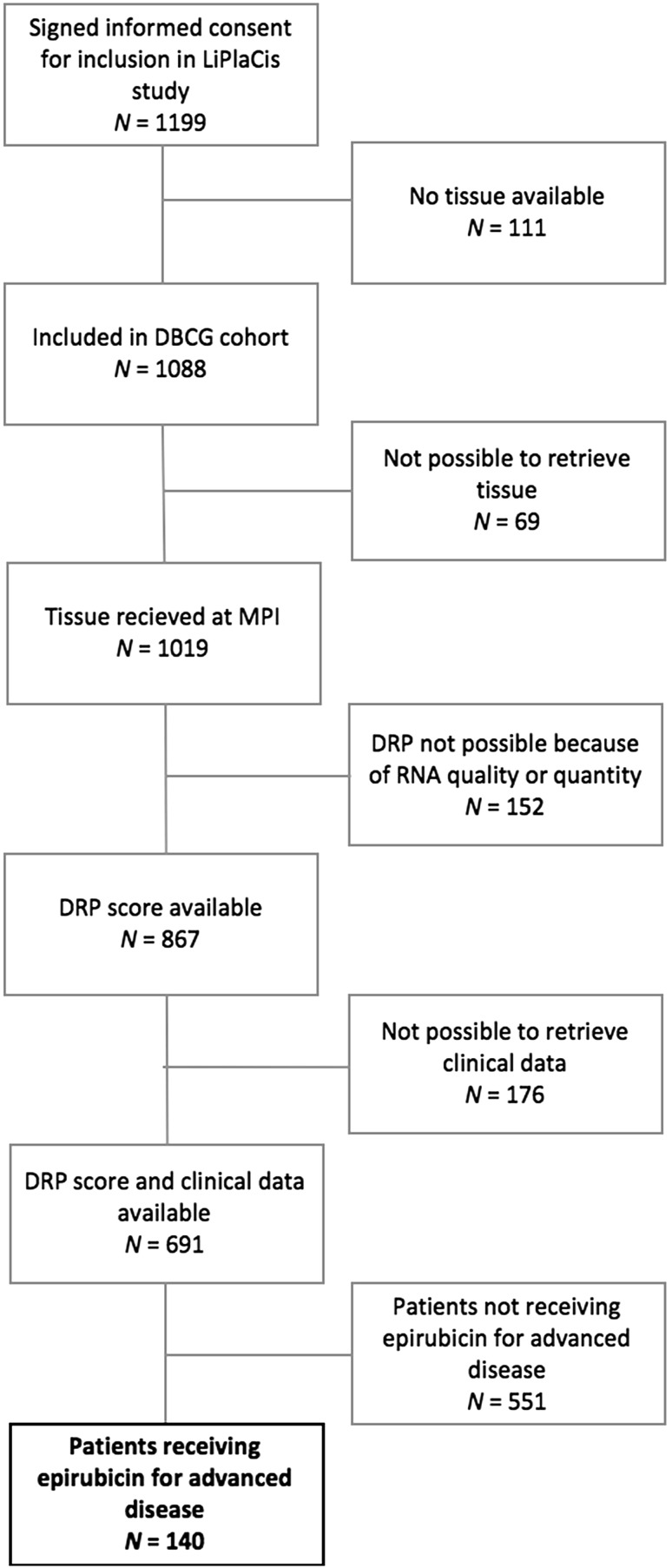
Flowchart of inclusion and exclusion

Data from all patients enrolled in the LiPlaCis study between April 2013 and November 2016 were obtained. Patients from the LiPlaCis cohort with a DRP score available and who received epirubicin as monotherapy in the advanced setting were identified for this study. Inclusion and exclusion flowchart are given in Fig. 2.

Clinical data were extracted retrospectively from patients’ medical and pathology records including information about primary tumor, metastases, and all treatments received in the adjuvant and advanced setting. Further, information regarding reason to change treatment was defined as either progression of disease, toxicity, long-lasting stable disease, physician or patient decision. The REMARK guidelines were followed where applicable [20].

The patients were followed by radiological assessment and clinical examination every 3–4 months, though a few patients had clinical progression and shifted treatment without radiological verification. Epirubicin was given as single-agent chemotherapy to all included patients.

#### Ethical approval

The LiPlaCis study (ID H-1-2013-016) and this substudy (ID HGH-2016-097) were approved by The Regional Committee on Health Research Ethics. All patients gave informed consent. Data in this substudy were collected in a retrospective manner and hence there were no health risks associated with the use of data. This was a non-intervention study and the results would not affect future treatment of the patients.

### Epirubicin sensitivity predictor (Epirubicin DRP) development

The in vitro-based method to develop a predictor of drug response has been described previously [14–18]. Briefly, it is an algorithm based on growth inhibition values (GI50) of the NCI60 cell lines [13] subjected to treatment with epirubicin. Gene expression measurements were performed with an Affymetrix HG-U133A array (Thermo Fisher Scientific, Waltham, MA, USA). After logit normalization, genes with a Pearson’s correlation coefficient to GI50 above 0.25 or below–0.25 were considered as potential biomarkers of sensitivity and resistance, respectively, to treatment and retained to contribute to the profile for epirubicin. 425 genes were correlated to sensitivity and 438 were correlated to resistance. To sort away genes only active in the in vitro setting, mRNA measurements from more than 3200 snap frozen clinical tumor samples were then applied to the profile. Hereby, only markers already known to be present in patient tumors contributed to the final profiles. The final signature consists of two sets of genes (158 up-regulated and 141 down-regulated features). The profile score was defined as the difference between the averages of the two groups of features. The scores were compared to a reference population of 819 breast cancer biopsies in order to obtain a percentile score for each patient sample.

### Tissue handling

Blocks of formalin-fixed, paraffin-embedded (FFPE) tumor tissue were sectioned from diagnostic biopsies from primary tumor from the enrolled patients as part of the LiPlaCis study. mRNA from the tissue was extracted, labeled, and hybridized to Affymetrix GeneChip 133 plus2 arrays (Thermo Fisher Scientific, Waltham, MA, USA) by Medical Prognosis Institute.

### Statistical analysis

Data were analyzed as specified in the statistical analysis plan. An additional supplementary analysis identifying a clinical cutpoint for the DRP was performed. The retrospectively collected clinical data were compared to the blinded predictions of sensitivity and resistance. All statistical prediction analyses were done blinded except for the supplementary analyses. Tests for interaction between the DRP and prior treatment were done to assess effect of these on the predictive value of the DRP.

Primary endpoint was time to progression (TTP) defined as time from start of treatment to progression. The statistical evaluation of TTP was done using the Cox proportional hazards model stratifying for treatment line. Patients not reaching progression were censored at the last seen date. The DRP was the explanatory variable and was entered into the model as a continuous covariate scored so that the hazard ratio (HR) was for a 50 percentage point difference.

A clinical cutpoint estimate for the DRP score was estimated assuming that the probability of progression at 6 months be at least 25%.

A multivariate model was performed including age, estrogen receptor (ER) status, number of metastatic sites, and performance status (PS) in addition to the DRP to adjust for factors of prognostic relevance. In addition, an adjustment for the year of treatment was considered. HR for both uni- and multivariate analyses is presented.

Model assessment of the linearity of the DRP covariate and the proportional hazards assumption were done using martingale residuals. Cases with missing values were not included in the analyses. A *P* value of < 0.05 (one sided) was considered significant.

All calculations and final database management were done using SAS (v9.4, Cary, NC, USA).

## Results

### Baseline patient characteristics

A total of 140 patients received epirubicin and were included in the analysis. The study population was diagnosed with primary BC between 1986 and 2015 and received epirubicin in the locally advanced or metastatic setting between May 1997 and November 2016. Of the 140 patients, four received epirubicin more than once in the metastatic setting besides the 20 patients treated with epirubicin in the adjuvant setting.

No associations were found between the epirubicin DRP and any of the clinical variables (Table 1).

**Table 1:** Baseline patient characteristics

Baseline characteristics	Received epirubicin in advanced setting	DRP association
	*N* = 140	*P* value
Age at first relapse, years in median (Q1–Q3)	62.1 (35.8–75.2)	*r* = 0.08^a^, *P* = 0.40
Time to relapse, years in median (Q1–Q3)	4.2 (0.0-25.2)	
ER status, *N* (%)		0.34
Positive	122 (87.1%)	
Negative	17 (12.1%)	
Data missing	1 (0.7%)	
HER-2 status, *N* (%)		0.31
Positive	16 (11.4%)	
Negative	110 (78.6%)	
Data missing	14 (10%)	
Adjuvant chemotherapy, *N* (%)		
CMF	9 (6.4%)	0.29
CEF	9 (6.4%)	0.84
EC-Tax	11 (7.9%)	0.91
Other	3 (2.1%)	
None	106 (75.7%)	
Data missing	2 (1.4%)	
Adjuvant anti-hormone therapy, *N* (%)		
Tamoxifen	18 (12.8%)	0.23
Tamoxifen + AI	33 (23.6%)	0.24
AI	15 (10.7%)	0.62
None	74 (52.8%)	
Data missing	–	
Adjuvant anti HER-2 directed therapy, *N* (%)		
Trastuzumab	5 (3.6%)	0.68
Data missing	31 (22.1%)	
Number of anti-hormone therapies prior to epirubicin, *N* (%)		
0 therapies	65 (46.4%)	
1	37 (26.4%)	
2	16 (11.4%)	
3	11 (7.9%)	
4 or more	11 (7.9%)	
Number of chemotherapies prior to epirubicin, *N* (%)		
0 therapies	62 (44.3%)	
1	38 (27.1%)	
2	25 (17.9%)	
3	7 (5.0%)	
4 or more	8 (5,7%)	
No. of treatment line receiving epirubicin, *N* (%)		
1	29 (20.7%)	
2	30 (21.4%)	
3	24 (17.1%)	
4	24 (17.1%)	
5 or more	33 (23.7%)	
Performance Status at time of treatment, *N* (%)		0.11
0–1	81 (57.9%)	
2	7 (5.0%)	
3 or more	2 (1.4%)	
Data missing	50 (35.7%)	
Number of metastatic sites in line receiving epirubicin, *N* (%)		0.13
1	57 (40.7%)	
2	36 (25.7%)	
3	27 (19.3%)	
4 or more	20 (14.3%)	
Place of metastatic sites in line receiving epirubicin, *N* (%)		
Breast	21 (15.0%)	
Lymph nodes	40 (28.6%)	
Skin	9 (6.4%)	
Liver	44 (31.4%)	
Bone or marrow	76 (54.3%)	
Lung or pleura	38 (27.1%)	
CNS	2 (1.4%)	
Peritoneal	8 (5.7%)	
Other	13 (9.3%)	

*ER* Estrogen receptor, *HER-2* human epidermal growth receptor 2, *CMF* cyclophosphamide, methotrexate and 5-fluorouracil, *CEF* cyclophosphamide, epirubicin and 5-fluorouracil, *EC-Tax*: epirubicin, cyclophosphamide and docetaxel, *AI* aromatase inhibitors

^a^Spearman rank correlation

Treatment was changed from epirubicin to another treatment due to either progression of disease (37.9%), physician decision (30%), toxicity (19.3%), because of long-lasting stable disease (3.6%), or patient decision (2.1%). Data are missing for ten patients. Median follow-up time was 6.2 months (range 1.5–13.2) and 50 patients (36%) had died at the last follow-up.

### The epirubicin gene expression profile

The Cox regression model, scoring the DRP as a continuous covariate, demonstrated that the DRP was significantly associated to TTP (*P* = 0.03, one sided). Median TTP was 9.3 months (95% confidence interval (CI) 7.2–13.2). Model validation did not demonstrate departures from the assumptions and results were not dependent on the chronological year of treatment (data not shown).

**Fig. 3:**
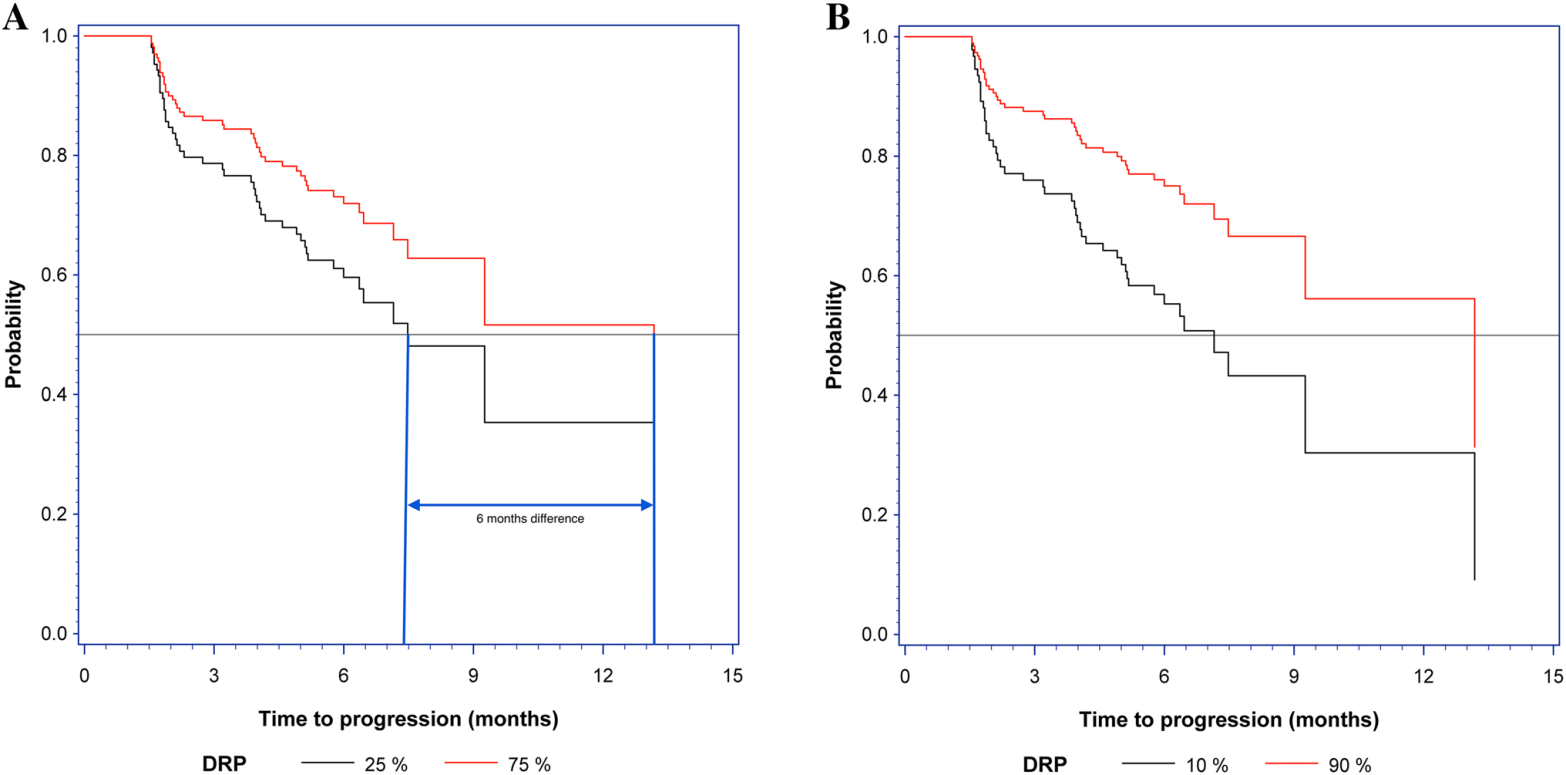
Cox regression with DRP-values of **a** 25 and 75% and **b** 10 and 90%. The gray horizontal line represents the median. In **a** the blue vertical lines point out the 6 months’ difference at median time to progression (TTP)

Based on the Cox regression comparing two patients with DRP scores differing by 50 percentage points, e.g., a DRP-value of 75% and a DRP-value of 25%, the estimated HR was 0.55 (95% CI –0.93, *P* = 0.03, one-sided). The estimated median TTP for a patient with a DRP-value of 75% was 13 months, whereas this was reduced to 7 months for patients with a DRP-value of 25%. This means that the difference between being predicted as a good responder (DRP ≥ 75%) versus a poor responder (DRP ≤ 25%) resulted in a difference in risk of progression of at least 12% at 6 months and the difference in median TTP was estimated to be 6 months as shown in Fig. 3a (blue lines).

Similarly, calculating for an 80 percentage point difference (DRP-value of 10% versus DRP-value of 90%), the HR was 0.39 (90% CI 0.17–0.89), demonstrating a strong separation in the risk between extreme DRP-values (Fig. 3b).

**Fig. 4:**
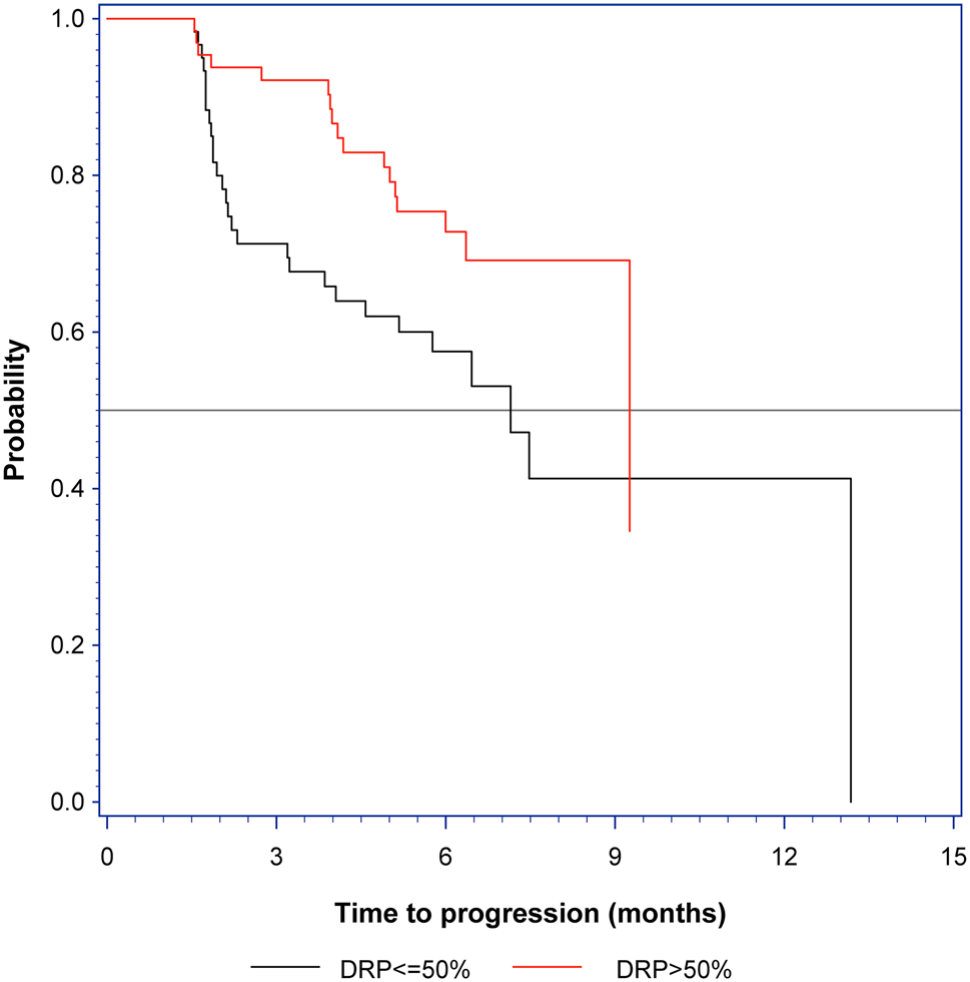
Kaplan Meier survival plot. The gray horizontal line indicates median survival time

The Kaplan Meier survival estimates are shown in Fig. 4. This analysis is done with DRP dichotomized at DRP = 50% and has a p value of 0.01 (one sided).

The DRP predicts time to death from initiation of treatment with epirubicin, HR 0.48 (90% CI 0.29–0.80), *P* = 0.009 (one sided).

### Multivariate analysis

The results from multivariate analysis are shown in Table 2. Reference age is 50–60 years (defined at start of epirubicin treatment), ER positive tumor, one metastasis and PS 0–1.

**Table 2:** Multivariate analysis

Parameter	Hazard ratio	Confidence interval	*P* value
DRP	0.57	[0.35–0.94]	0.032
Age			
< 50	0.47	[0.19–1.17]	
50–60	1.00 (Reference)	–	
60–70	0.54	[0.26–1.14]	
> 70	0.42	[0.18–0.97]	
ER negatives	4.49	[2.14–9.44]	0.0004
Metastatic sites			
1	1.00 (Reference)	–	0.07
2	1.89	[0.98–3.63]	
3	0.66	[0.27–1.63]	
> 3	0.64	[0.24–1.71]	
Performance status			
2 versus 0–1	2.74	[0.66–11.4]	0.36

It is well known that ER-negative tumors exhibit a more aggressive biology which is reproduced here although with few patients. No age dependence could be demonstrated. Finally, the tumor burden measured as number of metastases and PS were not statistically significant.

The analysis shows that only ER status is statistically significant in addition to the DRP.

The DRP is significant in both multi- and univariate analyses.

This retrospective study spans over almost 30 years. Including a covariate for the time from the primary diagnosis to the current treatment could not demonstrate a significant effect, *P* = 0.21 with the predictor remaining significant.

The DRP was not dependent on whether adjuvant or prior treatment with chemotherapy was given (Test for interaction, Cox regression model, *P* = 0.70).

A test for interaction between adjuvant or prior treatment with anthracyclines and the predictor was not significant (*P* = 0.69) though only 20 patients had received adjuvant anthracycline.

Prior taxane treatment can possibly lead to resistance to anthracycline treatment [21] but the interaction between treatment with taxanes either in the adjuvant setting or for advanced disease prior to the current treatment and the DRP was not significant in a multivariate model (*P* = 0.46).

### Supplementary analyses

The estimated clinical cut-off for progression at 6 months and with at least 25% with progression was a DRP-value of 77%. Patients with a DRP-value less than 38% had a probability of more than 25% of progression at 4 months.

## Discussion

We found a significant association of the continuous DRP with TTP in epirubicin-treated advanced BC patients. TTP was furthermore significantly longer in the high-DRP group (DRP ≥ 50) as compared to the low-DRP group.

The DRP for epirubicin has previously been evaluated on published material in a neoadjuvant setting in early BC with statistically significant findings [17]. This present study validates the former findings in an advanced BC cohort.

The applicability of an anthracycline biomarker in BC, e.g., topoisomerase II alpha (TOP2A) has previously been thoroughly reviewed but has not yet been introduced into clinical practice [22]. Today a biomarker for specific chemotherapy is still highly needed [23]. In order to assist the treating physician, the biomarker has to present proper clinical significance. Our supplementary analysis found that patients with a DRP of 77% or higher had a probability of 25% of at least 6 months before progression occurred. Further, we found that patients with a DRP-value of less than 38% had a probability of 25% of progression at 4 months. This means that the DRP possibly could predict which patients would benefit from epirubicin less or more than 6 months in an advanced setting.

Our results showed that the estimated median TTP for a patient with a DRP of 25% was 7 months compared to 13 months with a DRP of 75%. It could be argued that 7 months is not a bad result in advanced breast cancer. However, epirubicin is typically used early in the advanced setting and it is probably one of the most effective drugs which is corroborated by the fact that it is an important part of the standard adjuvant chemotherapy.

Previous validation of the DRP method has been clinical trials with selected populations not representing the broad diversity of patients. This study aimed to validate the DRP in an intention to treat analysis with non-selected all-comers which included all subtypes of BC supposedly representing the real world.

A strength of this study is the multicenter setup with ten major hospitals covering Denmark ensuring a broad clinical applicability, e.g., this setup did reduce the possible bias that different sites use epirubicin in different treatment lines.

The primary limitation to the study is the retrospective observational design.

Several of the included patients had their biopsy taken at time of primary diagnosis up to more than 20 years before our assays were done. These old biopsies were pooled with biopsies from patients diagnosed more recently resulting in a median age of biopsies of 10 years. However, date of biopsy did not affect prediction demonstrating that a fragile molecule like RNA can be extracted and measured more than a decade after biopsy. Biochemistry from FFPE tissue is notoriously difficult; however, methods are steadily improving and FFPE tissue may be a very valuable source of information in the future. To prevent possible bias from patients’ prior treatments such as taxane, in future studies it should be preferred to use new biopsies if possible instead of biopsies taken by primary diagnosis in case of long time gap between primary cancer and advanced disease.

The results from this study are remarkable and future studies predicting anthracycline response also as (neo)adjuvant therapy could be of interest. The DRP used in this setting could support the decision of potentially curable treatment compared to the palliative focus of this study.

The result from this study is clinically meaningful but could be further strengthened by a prospective study in a randomized setting between physician treatment decision and the DRP to demonstrate if such an approach will lead to increased overall survival. A prospective study with liposomal doxorubicin is already initiated using this technology in collaboration with DBCG.

In conclusion, this validation of the DRP showed significant prediction of response to anthracyclines in advanced BC. The DRP can support the clinician with a better-informed treatment decision for anthracyclines in advanced BC. In addition, the DRP can assist in development of more accurate treatment, prevent ineffective cytotoxic effects, and potentially impact patient survival.

## Data Availability

The datasets generated and analyzed during the current study are available from the corresponding author on reasonable request.
